# Epidemiological analysis of 3,219 COVID-19 outbreaks in the state of Baden-Wuerttemberg, Germany

**DOI:** 10.1017/S0950268821000911

**Published:** 2021-04-23

**Authors:** Aparna Dressler, Iris Finci, Christiane Wagner-Wiening, Martin Eichner, Stefan O Brockmann

**Affiliations:** 1 Department of Health Protection and Epidemiology, Baden-Wuerttemberg State Health Office, Stuttgart, Germany; 2 European Programme of Intervention Epidemiology Training (EPIET), Stockholm, Sweden; 3 Institute for Clinical Epidemiology and Applied Biometry, University of Tuebingen, Tuebingen, Germany

**Keywords:** Baden-Wurttemberg, COVID-19, Outbreaks, Setting, SARS-CoV-2, Surveillance

## Abstract

The severe acute respiratory syndrome coronavirus 2 (SARS-CoV-2) pandemic has emerged as an unprecedented global crisis challenging health systems. This paper aims to assess and characterise SARS-CoV-2 outbreaks in the state of Baden-Wuerttemberg to identify groups at greatest risk, to establish early measures to curb transmission. We analysed all mandatory notified (i.e. laboratory-confirmed) coronavirus disease (COVID-19) outbreaks with more than two cases in Baden-Wuerttemberg from calendar weeks 18–49 (from 27 April to 6 December 2020). We used the following classification for settings: asylum and refugee accommodation, care homes, care facilities, day care child centres, hobby-related, hospitality, hospitals, households, other, residence halls, schools, supported housing, training schools, transportation, treatment facilities and workplace (occupational). We used R program version 3.6.3 for analysis. In our analysis, 3219 outbreaks with 22 238 individuals were included. About 48% were in household and hobby-related settings. Care homes accounted for 9.5% of outbreaks and 21.6% of cases. The median age across all settings was 43 (interquartile range (IQR) 24–63). The median age of cases in care homes was 81 (IQR 56–88). Of all reported cases in care homes, 72.1% were women. Over 30% (466/1511) of hospitalisations were among cases in care homes compared to 17.7% (268/1511) in households. Overall, 70% (500/715) of all deceased persons in outbreaks in the study period were in care homes compared to 4.2% in household settings (30/715). We observed an exponential increase in the number of notified outbreaks starting around the 41st week with *N* = 291 outbreaks reported in week 49. The median number of cases in outbreaks in care homes and care facilities after the 40th week was 14 (IQR 5–29) and 11 (IQR 5–20), respectively, compared to 3 (IQR 3–5) in households. We observed an increase in hospitalisations, and mortality associated with COVID-19 outbreaks in care homes after the 40th week. We found the care home demographic to be at greatest risk after the 40th week, based on the exponential increase in outbreaks, the number of cases, hospitalisations and mortality trends. Our analysis highlights the necessity of targeted, setting-specific approaches to control transmission in this vulnerable population. Regular screening of staff members and visitors' using rapid antigen point-of-care-tests could be a game-changer in curbing transmission in this setting.

## Background

The severe acute respiratory syndrome coronavirus 2 (SARS-CoV-2) pandemic is an unprecedented global crisis challenging health systems. Similar to in most countries in the European Union, the German regional and national response shifted from an initial containment strategy, to a control strategy which aims to strike a balance between imminent public health considerations and economic considerations and allow as much social, economic, educational and cultural life as possible to take place. To curb the rapid spread of the SARS-CoV-2 pandemic in Germany, the Federal State Governments declared two lockdowns. Currently, in the state of Baden-Wuerttemberg (BW), a second hard lockdown is underway, which came into effect on the 16th of December 2020.

Here, we briefly describe some of the pandemic control measures implemented before and in the study period. The first lockdown in Baden-Wuerttemberg came into force on the 17th of March 2020. Various measures were introduced to stem the spread of the infection, including closing universities, schools and day-care centres. Emergency childcare was possible if parents were critical infrastructure workers. Cultural facilities, cinemas, pools, public and private sport facilities such as gyms, dance schools, amusement parks, zoos, etc. were closed. Restaurants were allowed to open provided a distance of at least 1.5 meters was guaranteed between tables. To protect particularly vulnerable people, with some exceptions, visitors were not allowed in facilities such as care homes. Individuals that visited high-risk areas abroad or particularly affected regions in Germany in the last 14 days, or those who were in contact with an infected person or who displayed symptoms of a respiratory infection were prohibited from entering facilities such as schools and day care centres.

Over time, various regulations in Baden-Wuerttemberg were enacted and updated to calibrate measures, including reopening schools, day care centres, retail and the hospitality sector, based on the epidemiological situation. Retail was permitted for shops with a minimum sales area of 800 square meters from the 20th of April 2020. On the 16th of June 2002, an updated regulation of the state government allowed schools, including elementary schools and kindergartens, to open under strict distancing and hygiene rules. On the 23rd of June 2020 a new regulation of the state government entailed various recommendations and mandated wearing a non-medical everyday mask or a comparable mouth and nose cover when using public transport, in waiting areas airport buildings and in medical facilities as non-medical settings such as at the hairdressers, massage and cosmetic studios. Occupational safety guidelines were specified, including hygiene practices which required employers to provide masks for employees.

On the 1st of November 2020, an updated regulation of the state government required retail establishments and markets to limit the number of customers present at the same time to a maximum of one or one per 10 square meters of sales area. For shops that were smaller than 10 square meters, a maximum of one customer was permitted. A partial lockdown (lockdown light) came into effect on the 2nd of November 2020. Further restrictions were imposed, such as only members of one's own household and another household could meet in public. Restaurants, bars etc. were closed, however, pick-up and delivery of food was allowed. Non-essential travel was discouraged. Day care centres, schools and other educational institutions as well as retail remained open. Due to rising infections despite the partial lockdown, further restrictions were imposed and a hard lockdown came into effect on the 16th of December 2020 (51st week).

The dynamic nature of the pandemic warrants ongoing characterisation and assessment of outbreak settings to identify groups at greatest risk and settings where transmissions are occurring, to establish early measures to curb transmission. The current analysis aims to assess and characterise SARS-CoV-2 outbreaks in Baden-Wuerttemberg. Our analysis provides a reference for decision makers to formulate and adjust control measures.

## Methods

We analysed all mandatory notified (i.e. laboratory-confirmed) coronavirus disease (COVID-19) outbreaks from Baden-Wuerttemberg in calendar weeks 18–49 (27th of April to 6th of December 2020). COVID-19 cases are notified to the local public health department in the respective districts, in accordance with the German Protection against Infection act (IfSG). The data are then transferred to the respective federal state health authority. Laboratory confirmation requires the detection of SARS-CoV-2 nucleic acid by polymerase chain reaction (PCR) testing. In the following analysis, the term ‘COVID-19’ covers SARS-CoV-2 infections as well as cases of COVID-19 that were PCR test confirmed. In this analysis, we included all outbreaks with more than two cases reported by a local public health authority that occurred within an epidemiological context [1]. New settings (categories) emerged over the course of the pandemic. We used the following classification for settings: asylum and refugee accommodation, care homes, care facilities, day care child centres, hobby-related, hospitality, hospitals, households, other, residence halls, schools, supported housing, training schools, transportation, treatment facilities and workplace (occupational) (Table 1 and Supplementary TableS1). Epidemiological characteristics of outbreaks were descriptively analysed. We used R program version 3.6.3 for analysis [2].

**Table 1.** COVID-19 outbreaks in the state of Baden-Wuerttemberg in calendar weeks 18–49

Setting	Outbreaks (*N*, %)	Total cases (*N*, %)	Cases in outbreaks (median, IQR)	Age (*y*, median, IQR)	Hospitalised (*N*, %)	Deceased (*N*, %)
Asylum accommodation	53 (1.6)	603 (2.7)	8 (4–14)	26 (17–34)	13 (0.9)	1 (0.1)
Care facilities	59 (1.8)	826 (3.7)	6 (3–15.5)	59 (40–83)	71 (4.7)	45 (6.3)
Care homes	307 (9.5)	4801 (21.6)	9 (4–23)	81 (56–88)	466 (30.8)	500 (70)
Day care centres	61 (1.9)	343 (1.5)	4 (3–7)	29 (5–45)	2 (0.1)	0 (0)
Hobby-related	165 (5.1)	1484 (6.7)	5 (3–7)	28 (19–44)	45 (3)	3 (0.4)
Hospitality	48 (1.5)	251 (1.1)	4 (3–6)	30 (20–49.5)	9 (0.6)	0 (0)
Hospitals	87 (2.7)	875 (3.9)	3 (3–10)	51 (20–49.5)	300 (19.9)	47 (6.6)
Households	1367 (42.5)	5830 (26.2)	4 (3–5)	34 (18–50)	268 (17.7)	30 (4.2)
Residence halls	12 (0.4)	118 (0.5)	6.5 (4–10)	21 (16–31.8)	0 (0)	0 (0)
Schools	91 (2.8)	511 (2.3)	4 (3–6)	16 (11–32)	3 (0.2)	0 (0)
Supported housing	104 (3.2)	656 (3)	3.5 (3–5)	45 (26–68)	35 (2.3)	23 (3.2)
Training schools	12 (0.4)	94 (0.4)	4.5 (3–7.6)	16 (11.2–31)	2 (0.1)	0 (0)
Transportation	8 (0.2)	37 (0.2)	5 (3–5)	36 (20–60)	3 (0.2)	0 (0)
Treatment facilities	47 (1.5)	335 (1.5)	5 (3–8)	58 (39.5–74)	73 (4.8)	10 (1.4)
Workplace	232 (7.2)	2184 (9.8)	5 (4–9)	40 (28–53)	78 (5.2)	8 (1.1)
Other	175 (5.4)	1012 (4.6)	4 (3–5)	33 (21–52)	42 (2.8)	4 (0.6)
Unknown	391 (12.1)	2278 (10.2)	4 (3–6)	39 (21–56)	101 (6.7)	44 (6.2)
Total	3219 (100)	22 238	4 (3–6)	43 (24–63)	1511 (100)	715 (100)

Accommodation for asylum seekers including refugees; care facilities (for the disabled or other individuals in need of care); care homes include day care centres for senior citizens and long-term care homes for the aged; hobby-related settings include, camping and forest and club membership; hospitality settings include, hotels, restaurants, diners, inns and hostels; supported housing (includes lodging in a dwelling, as well as housing with support, supervision or care for older people, people with disabilities, mental health issues etc.); workplace i.e. occupational settings (excluding hospitals, day care centres and schools); residence halls (for students, this category also includes children's homes and juvenile homes); training schools (educational institute or training centres); treatment facilities include rehabilitation centres and medical practices.

## Results

**Fig. 1.**
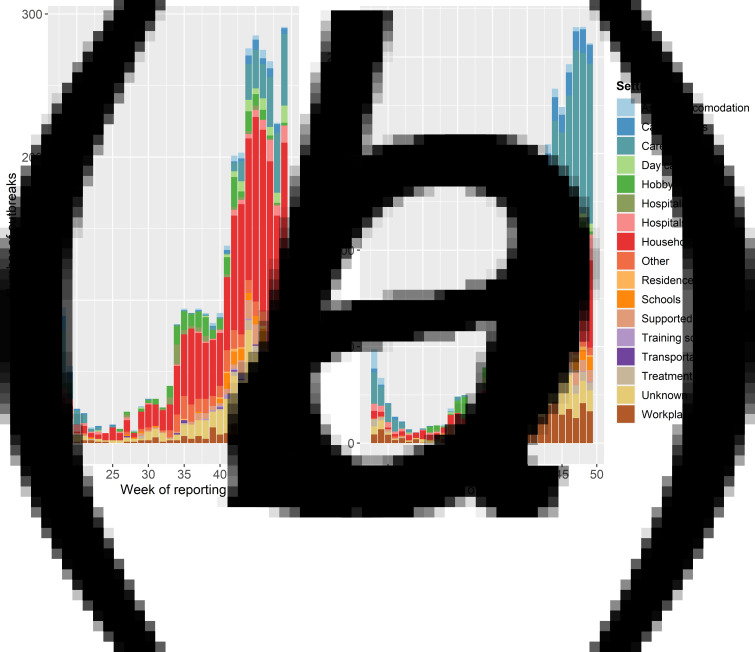
COVID-19 outbreaks in BW in calendar weeks 18–49, 2020 (a) by setting and reporting week of outbreaks; (b) number of cases in outbreaks by reporting week (3219 outbreaks; 22 238 cases).

We included 3219 outbreaks with 22 238 individuals over a 32-week period (calendar weeks 18–49) (Table 1, Fig. 1a). About 48% of all outbreaks occurred in household and hobby-related settings (Table 1 and Fig. 1). We recorded 9.5% of all outbreaks in care homes, which accounted for >20% of all cases in outbreaks in the current analysis. We did not have information on the setting in 12.1% (*N* = 391) of outbreaks. Over 30% (466/1511) of hospitalisations were among cases in care homes compared to 17.7% (268/1511) in households. Overall, 70% (500/715) of all deceased persons in outbreaks in the study period were in care homes compared to 4.2% in household settings (30/715). We observed a shift in the frequency of outbreaks and their settings over time. After an initial decrease in outbreaks from calendar week 18 (*N* = 95) until the 26th week (*N* = 10), an increase in the number of outbreaks was observed from the 30th week (*N* = 32) until the 35th week (*N* = 94). We observed an exponential increase in the number of notified outbreaks starting around the 41st week (*N* = 138) with *N* = 291 outbreaks reported in week 49.

**Fig. 2.**
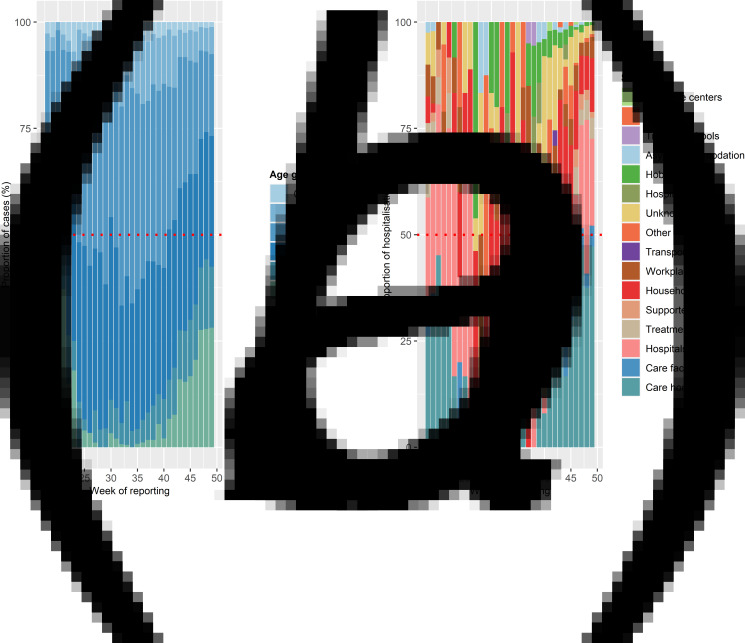
(a) Proportion of COVID-19 cases in outbreaks (3219 outbreaks; 22 238 cases) in BW, in calendar weeks 18–49, 2020 by age group; (b) Proportion of hospitalisations by reporting week in all reported settings.

**Fig. 3.**
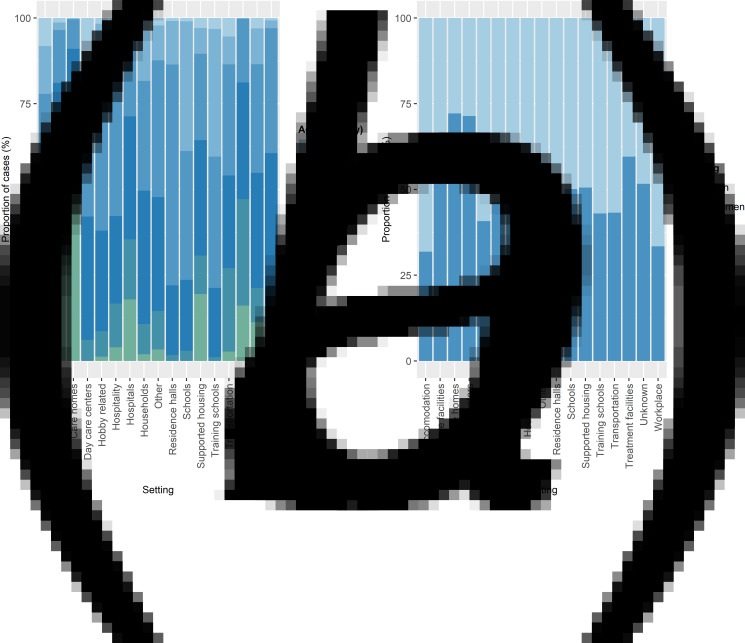
(a) Age and (b) sex distribution across settings with COVID-19 outbreaks (3219 outbreaks; 22 238 cases) in BW, in calendar weeks 18–49, 2020.

**Fig. 4.**
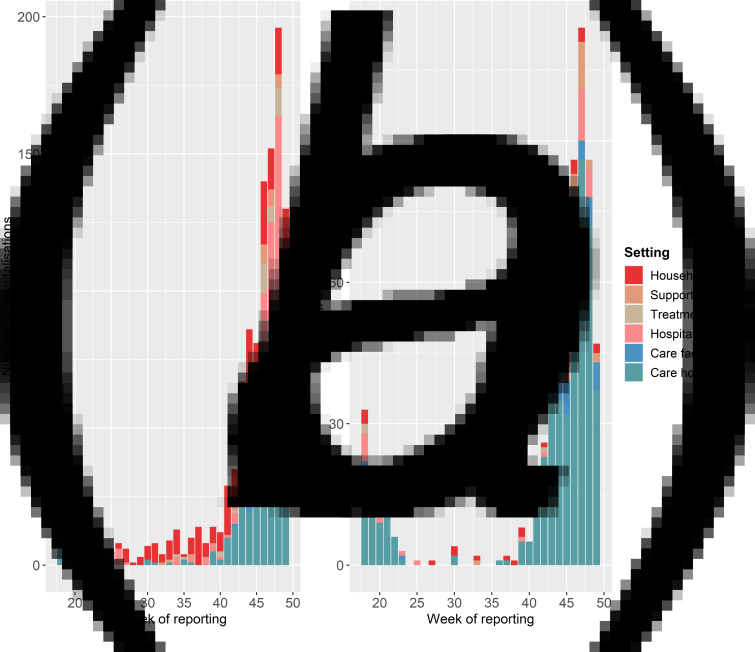
(a) Number of hospitalisations and (b) number of deaths due to or with COVID-19 in outbreaks in selected settings by reporting week in BW, in calendar weeks 18–49, 2020 (see Table 1 for further details).

Over the 32 week period, we observed an altering age distribution in outbreaks (Fig. 2). More outbreaks occurred in care homes initially, followed by a shift in outbreaks to household settings and hobby-related settings which steadily increased around the 30th week (Fig. 1b). The number of outbreaks in care homes stabilised at a low level of outbreaks between weeks 24 and 40, but increased thereafter. Figures 3a and b illustrate the age and sex distribution per setting. The number of cases, hospitalisations and deaths also increased over time after the 40th week, particularly in care home settings (Figs 1, 2b and 4). The median number of cases in outbreaks in care homes and care facilities after the 40th week was 4 (interquartile range (IQR) 5–29) and 11 (IQR 5–20), respectively, compared to 3 (IQR 3–5) in households.

## Discussion

We observed, a somewhat stepwise pattern of increase in outbreaks, with temporary stabilisation in certain periods. We observed a substantial increase in the number of outbreaks around the 41st week. To contain the rapid spread of SARS-CoV-2, two countrywide lockdowns were imposed in Germany. The first lockdown in Baden-Wuerttemberg came into effect on the 17th of March 2020 (12th calendar week). A stepwise reopening followed around the 20th of April 2020 (17th calendar week). A second, partial lockdown in Baden-Wuerttemberg went into effect on the 2nd of November 2020 (45th week in our analysis), which resulted in the closure of bars and restaurants, but left all shops open. In lieu of rising infections, further restrictions were imposed and a hard lockdown came into effect on the 16th of December 2020 (51st week).

The pattern of outbreaks altered over time. The primary site of SARS-CoV-2 outbreaks from around the 30th calendar week was in private settings i.e. household and hobby-related settings. In such settings, suboptimal or no adherence to preventative measures constitutes a major risk factor, possibly, due to a low risk perception [3]. The median number of cases per outbreak across settings has increased in care homes and facilities as compared to households after the 40th week. This suggests that although outbreaks continue to occur in household settings at a high frequency, they are low amplitude outbreaks i.e. with few people. In Baden-Württemberg, since the beginning of the pandemic, COVID-19 outbreak investigations entailed rigorous testing of symptomatic cases and their contacts, by PCR. However, on 9th November 2020 i.e. week 46, a new SARS-CoV-2 test strategy in Baden-Wuerttemberg was approved which entailed using antigen-point-of-care-tests (POCTs) for screening in specific settings such as care home residents and staff, medical personnel and patients in medical settings, etc. Those that tested positive underwent confirmatory PCR testing. The POCTs may have resulted in detecting asymptomatic cases and under the radar outbreaks. However the uptake of these tests was slow and given the timeline of our analysis and the introduction of these tests, we do not expect POCTs in specific settings to have significantly influenced outbreak detection. In general, in our assessment, it is unlikely that the trends reported in the paper reflect an altered access to diagnostic testing in outbreak investigations over time.

Larger outbreaks are occurring in specific ‘high-risk’ settings such as care homes. Residents of long-term care facilities (LTCFs i.e. care homes and care facilities) are a medically and socially vulnerable group with an elevated risk of severe disease and death due to COVID-19 [4]. For COVID-19, there are strong indications of age dependence in severity and mortality [5, 6]. Community transmission of SARS-CoV-2 in Baden-Wuerttemberg is at a high level. The 7-day incidence as of 20 December 2020 (12:00 AM) was 204 per 100 000 [7]. This has serious implications for vulnerable populations exposed to care givers/personnel that might be ‘silent shedders’ who might spread the disease unawares in care homes and hospitals. Given the lack of effective therapeutics and the slow rollout of licensed vaccines, non-pharmacological public health measures are the best interventions that exist at this point in time against the pandemic. Even after the pace of vaccinations picks up, it is likely that there will be a considerable period of time where non-pharmacological public health interventions will remain the mainstay of prevention until herd immunity is achieved. A combination of adherence to stringent hygiene guidelines, supplemented by mandatory rapid antigen POCTs [8] to enhance testing, tracing and isolation of suspected COVID-19 cases and their contacts, in high-risk settings might be a game-changer. In care homes, these tests if used in a timely and appropriate fashion, with regular screening of staff members and visitors, could prove to be instrumental in preventing or interrupting disease transmission. That said, rapid antigen POCTs for SARS-CoV-2 have a lower sensitivity and specificity than the PCR test and perform best when the viral load is generally highest [8]. Antigen levels in specimens collected before symptom onset, or late in the course of infection, may be below the antigen test's limit of detection. This can lead to false-negative test results. The PCR test remains the ‘gold standard’ for clinical diagnostic detection of SARS-CoV-2. A positive antigen POCT requires PCR-confirmation according to current recommendations in Germany. Serial testing at close time intervals could quickly identify someone with a SARS-CoV-2 infection and prevent further transmission particularly in closed congregate settings such as care homes. Novel non-invasive gargle or spit tests may contribute to a higher acceptance and willingness to test as compared with tests requiring nasal and throat swabbing [9].

Our data underscore the need to focus on ‘protection’ of vulnerable population groups in high-risk settings. We recorded numerous outbreaks in various settings over the 32 week period, which highlights the continued need for the entire population to be committed to infection prevention and control. Currently, data on adherence to COVID-19-related personal safety guidelines such as social distancing, and mask wearing, in Baden-Wuerttemberg are not available. We found relatively few outbreaks in day care centres and schools in the 32 week period. Furthermore, outbreaks in these settings had few cases. A higher proportion of outbreaks and cases in schools was observed among older age groups (³15 years of age), including staff members or other adults linked to the outbreak. A previous analysis on surveillance data from Germany also made similar observations on infections in schools [10].

The limitations of the current analysis must be considered when interpreting the results. First, surveillance data give an indication of the trends of infection in the population, based on primarily symptomatic subjects and their contacts. However, an estimated 40–45% of SARS-CoV-2 infections in adults are asymptomatic [11]. It is possible that some outbreaks in certain settings such as day care centres and schools, may not have been detected because of asymptomatic infections, however, children may also have a lower susceptibility to infection, in addition to a lower propensity to show clinical symptoms [12]. A recent German study found a high seroprevalence in children compared to health-authority reported cases [13]. However, one cannot exclude the presence of false positives due to beta coronavirus cross-reactivity in this study [13]. Second, there may be a time lag in outbreak notifications due to information gathering processes at the level of the notifying local health offices. Third, the quality of reporting in the national surveillance system varies in terms of completeness of data. Furthermore, there may be inconsistencies and delays in recording individual cases belonging to a specific outbreak. Fourth, despite the multitude of choices of settings available in the surveillance software in use, it is not always possible to reliably determine the setting where the actual infection transmission occurred. Outbreaks may be under-recorded in certain settings such as public transportation, particularly, because infections could not be identified and potential contacts might be difficult to trace.

## Conclusions

Our analysis found a substantial increase in outbreaks, particularly in care homes from weeks 41 to 49. On the basis of the number of cases, hospitalisation and mortality trends, we found the care home demographic to be at greatest risk. Our analysis highlights the necessity of targeted, setting-specific approaches to control transmission in this vulnerable population. In care homes, regular screening of staff members and visitors' using rapid antigen POCTs could be instrumental in curbing transmission.

## Data Availability

The readers can contact the corresponding author if they want access to the data used in the current study.

## Supplementary material

For supplementary material accompanying this paper visit http://dx.doi.org/10.1017/S0950268821000911. click here to view supplementary material
