# Supplementary material for: Epidemiological analysis of 3,219 COVID-19 outbreaks in the state of Baden-Wuerttemberg, Germany
Source: Epidemiol Infect. 2021 Apr 23;149:e101. doi: 10.1017/S0950268821000911 (PMC8111206; doi:10.1017/S0950268821000911)
Supplement: Supplementary file 1 [file S0950268821000911sup001.docx]

**Supplementary Material**

**Supplementary Table S1.** **COVID-19 outbreaks in the state of Baden-Wuerttemberg in calendar weeks 18 to 49 with row percentages**

| Setting | Total Cases  (N, %) | Hospitalized  (N, %) | Deceased  (N, %) |
| --- | --- | --- | --- |
| Asylum accommodation | 603 (100) | 13 (2.2) | 1 (0.2) |
| Care facilities | 826 (100) | 71 (8.6) | 45 (5.5) |
| Care homes | 4,801 (100) | 466 (9.7) | 500 (10.4) |
| Day care centers | 343 (100) | 2 (0.6) | 0 (100) |
| Hobby related | 1,484 (100) | 45 (3) | 3 (0.2) |
| Hospitality | 251 (100) | 9 (3.6) | 0 (100) |
| Hospitals | 875 (100) | 300 (34.3) | 47 (5.4) |
| Households | 5,830 (100) | 268 (4.6) | 30 (0.5) |
| Residence halls | 118 (100) | 0 (100) | 0 (100) |
| Schools | 511 (100) | 3 (0.6) | 0 (100) |
| Supported housing | 656 (100) | 35 (5.3) | 23 (3.6) |
| Training schools | 94 (100) | 2 (2.1) | 0 (100) |
| Transportation | 37 (100) | 3 (8.1) | 0 (100) |
| Treatment facilities | 335 (100) | 73 (21.8) | 10 (3) |
| Workplace | 2,184 (100) | 78 (3.6) | 8 (0.4) |
| Unknown | 2,278 (100) | 101 (4.4) | 44 (1.9) |
| Other | 1,012 (100) | 42 (4.2) | 4 (0.4) |
| Accommodation for asylum seekers including refugees; care facilities (for the disabled or other individuals in need of care); care homes include day care centers for senior citizens and long-term care homes for the aged; hobby related settings include, camping and forest, and club membership; hospitality settings include, hotels, restaurants, diners, inns, and hostels; supported housing (includes lodging in a dwelling, as well as housing with support, supervision or care for older people, people with disabilities, mental health issues etc.); workplace i.e., occupational settings (excluding hospitals, day care centers, and schools), residence halls (for students, this category also includes children’s homes and juvenile homes); training schools (educational institute or training centers); treatment facilities include rehabilitation centers and medical practices. | | | |
